# Supplementary material for: “Grafting-To” Covalent Binding of Plasmonic Nanoparticles onto Silica WGM Microresonators: Mechanically Robust Single-Molecule Sensors and Determination of Activation Energies from Single-Particle Events
Source: Sensors (Basel). 2023 Mar 25;23(7):3455. doi: 10.3390/s23073455 (PMC10098601; doi:10.3390/s23073455)
Supplement: Supplementary file 1 [file sensors-23-03455-s001.zip › Supplementary Material.pdf]

Supplementary Material

Article

"Grafting-To" Covalent Binding of Plasmonic Nanoparticles onto Silica WGM Microresonators: Mechanically Robust Single-Molecule Sensors and Determination of Activation Energies from Single-Particle Events

Mariana P. Serrano [1],* , Sivaraman Subramanian [2], Catalina von Bilderling [1], Matías Rafti [1] and Frank Vollmer [2],*

[1] INIFTA-CONICET, Departamento de Química, Facultad de Ciencias Exactas, Universidad Nacional de La Plata, La Plata B1900, Argentina

[2] Living Systems Institute, Department of Physics & Astronomy, University of Exeter, Exeter EX4 4QD, UK

* Correspondence: marianaserrano@quimica.unlp.edu.ar (M.P.S.); f.vollmer@exeter.ac.uk (F.V.)

1) Au- Nanoparticles characterization

Average Length (TEM): 45.5 ± 6.3 nm (JEOL 1010 Transmission Electron Microscope)

Mass Concentration (Au): 0.031 mg/mL (Thermo Fisher X Series 2 ICP-MS)

CV, Length: 13.9 %

CV, Width: 6.7 %

Particle Concentration: 2.36E+11 parts/mL

Average Width (TEM): 17.4 ± 1.2 nm

Hydrodynamic Diameter: 13 nm (Malvern Zetasizer Nano ZS.)

Zeta Potential: -67 mV (Malvern Zetasizer Nano ZS.)

Average Aspect Ratio: 2.6

% Absorbance at 660 nm: 97.4 % (Agilent 8453 UV-Visible Spectrometer)

OD at 660 nm: 1.06

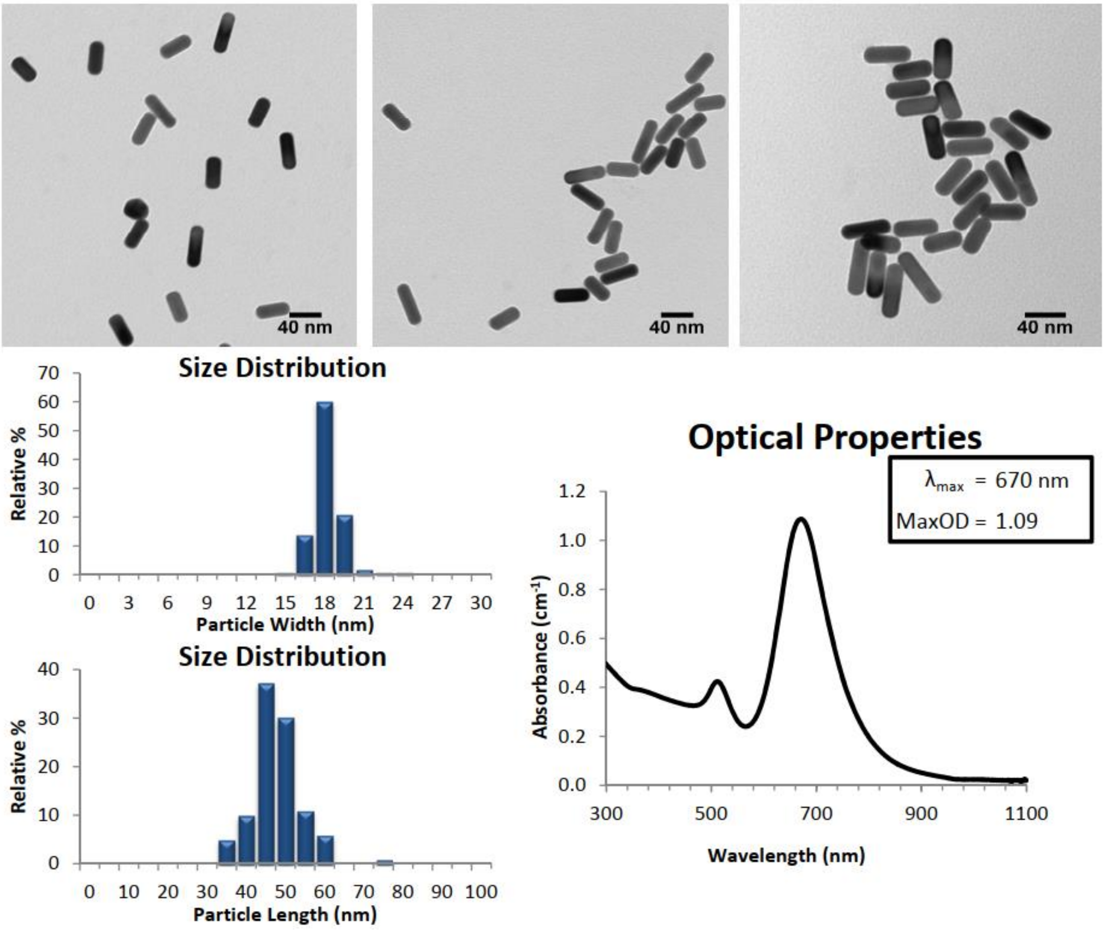

Figure S1.a TEM images, relative size distribution analysis and UV-vis as provided by supplier (nanoComposix-San Diego CA, US).

Modification was performed following a procedure introduced recently by Liz-Marzán et al. (DOI: 10.1021/acs.chemmater.8b04647, Chem. Mater. 2019, 31, 57−61) in which silanization reactions were explored for creation of core-shell architectures from gold nanoparticles and morphologies of the starting materials were proven to be preserved. The setup hereby employed uses laser wavelengths which are compatible with plasmon resonance of the NR employed, thus if the modification procedure would alter such feature such interaction should be affected, and single-particle events should not be detected. Unfortunately, the concentrations used for modification procedure are highly unpractical for such determination post-modification, and therefore we resorted to AFM rather as a confirmation tool, having in mind the information provided. Actually, the ex-situ

determined size and shape is a good indicator not only of the presence of the NR but also of the successful grafting that can be achieved with silica-based materials. We did performed UV-vis experiments for ensuring conservation of NR plasmonic shift, which we show below.

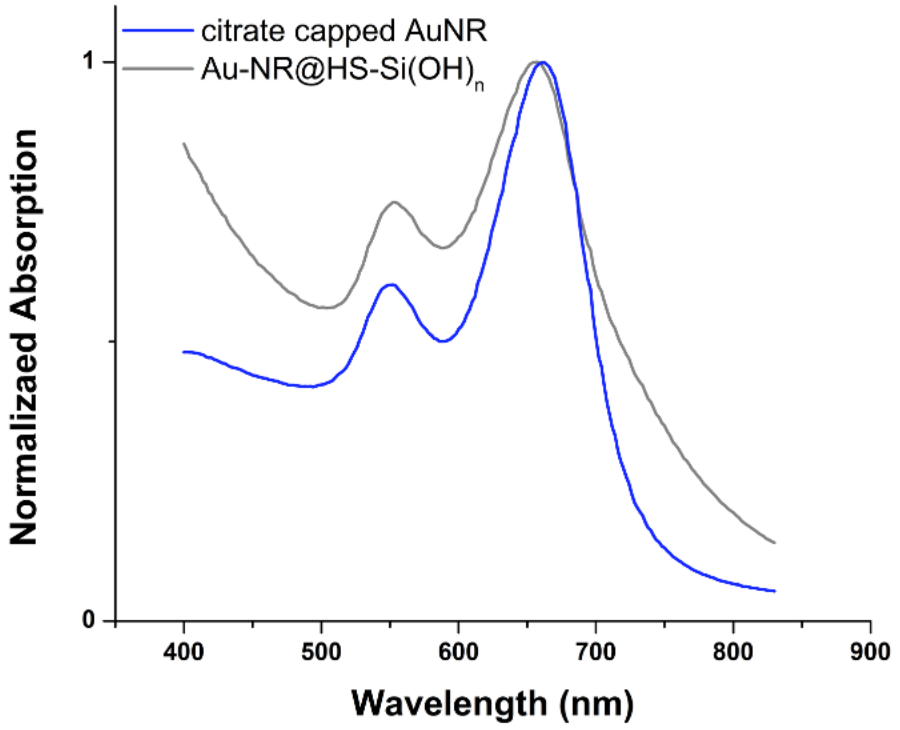

Figure S1.b Plasmonic shifts for the nanorods employed before and after carrying silanization reactions.

2) Protocols employed for DNA hybridization using MPTMS

Colloidal modification of Citrate-capped Au-NR with MPTMS

Stock 0.25 % v/v ethanolic MPTMS solutions were prepared from pure MPTMS (d=1.050 g ml^{-1}, 95% p/p) and stored in dry and cool conditions for subsequent use in colloidal modification of citrate capped AuNR (25 µL MPTMS in 1000 µL final volume completed with milliQ water). The procedure followed for preparing 100 µL of MPTMS-modified AuNR concentration 0.5 OD, hereafter referred as Au-NR@HS-Si(OH)n, can be described as follows; 48 µL of miliQ water were added to 50 µL citrate-capped AuNR (NanoXact, concentration 1.0 OD); after brief mixing using ultrasonic bath, 2 µL of a (1:10) dilution in milliQ water of MPTMS stock solution were incorporated under magnetic stirring and left to react for 40 minutes (the procedure results in a 1:1000 molar ratio of AuNR:MPTMS, much lower than what earlier reported to produce thick silica coatings in core-shell AuNR@silica synthesis protocols). The conditions described are known to cause partial hydrolysis of MPTMS ethoxy moieties, thus yielding silanol groups exposed oriented outwards Au-NR surface. This can be understood by considering oxidative chemisorption occurring upon Au-thiol interactions, which replace the relatively weakly bounded citrate capping agent, as extensively demonstrated in recent literature. The fact that Au-NR@HS-Si(OH)n remain well-dispersed and that the above described 660 nm centered wavelength plasmon adsorption band is preserved, is in line with the presence of highly hydrophilic silanol moieties. Care must be taken regarding the pH of solutions and buffer solutions used, e.g., it is known that strongly alkaline conditions (pH≥10) and the addition of ammonia produces the catalysis of condensation reactions leading to the formation of silica shell.

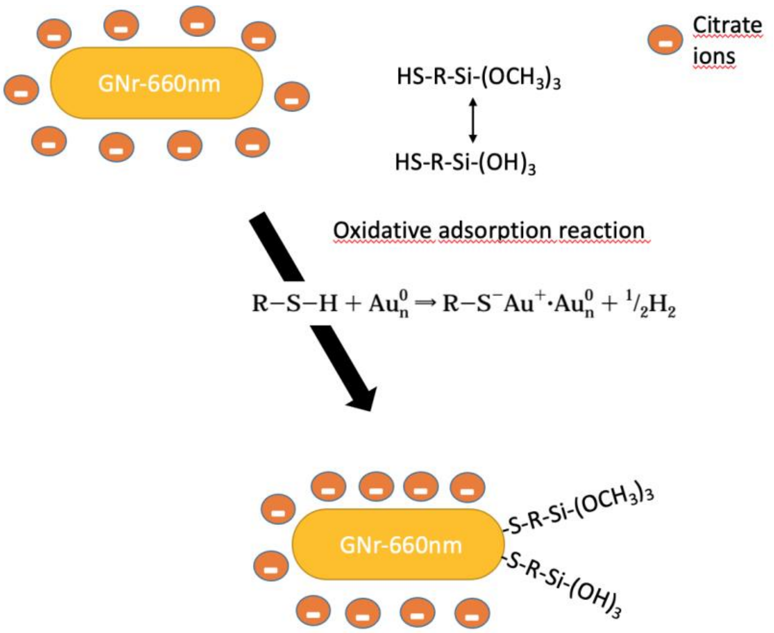

Figure S2. Schematics of the reaction between MPTMS and citrate capped Au-NR. (See Liz-Marzán et al. Chem. Mater. 2019, 31, 57-61, and Ulman Chem. Rev., 1996, 96, 1533-1554)

Colloidal modification of Citrate-capped Au-NR with MPTMS and hybridization with complementary DNA

First step of modification includes activation of DNA with TCEP. The details sequence for the thiolate single stranded-DNA correspond to SS-A9-DNA: 5'-SH-AAAAAAAAAGTTATGTTATGT-3' and 5'-ACATAACATAAC-3' for its full complementary (C12). The 9-adenine residues are meant to generate A-A complex which would facilitate interaction with Au-NR surface. Mix 20 µL of A9 DNA which is stored in the freezer (0,1 mM, for a total of 2 nanomoles, aliquots are already separated), with 10 µL of TCEP solution (10 mM concentration, for a total 100 nano-moles, look for the solution in the normal fridge). Resulting (TCEP:A9) ratio is (50:1), this should cleave the S-S bond thus activating the thiolated DNA A9. Add 5 µL pH 5 sodium citrate 100 mM (for 100 ml buffer, mix Sodium Citrate dihydrate (mw: 294.1 g/mol) 1.697 g and Citric Acid (mw: 192.1 g/mol) 813 mg). Mix in ultrasonic bath for 1 min, and leave to react for 90 min. This procedure yields activated A9-DNA. Second step consist of Au-NR functionalization using activated A9-DNA. Take 0.5

µL of the above prepared A9-DNA solution (0.02857 nanomoles) and 100 µL of citrate capped as-received Au-NR (1 OD, 1.91 10^{-5} nanomoles, for a final ratio (A9-DNA:Au-NR) of (1:1500) approximately), then mix in ultrasonic bath thermostatic 40 °C for 60 min. This step allows surface positioning of thiol-terminated activated A9-DNA. Then add 2 µL citrate buffer pH 3 500 mM (for 10 ml buffer, mix 8.2 ml citric acid 500 mM plus 1.8 ml citrate 500 mM), mix in ultrasonic bath for 1 min and leave to react in thermostatic bath for 30 min. This yields DNA hybridized nanorods (A9-DNA-Au-NR).

Third step is the reaction of the above prepared NR with MPTMS in order to confer affinity towards silanol moieties on the surface of the microresonator. Add to the above A9-DNA-Au-NR colloidal suspension 2 µL of the MPTMS solution prepared as described in the previous protocol ((1:10) dilution in milliQ water of stock 0.25 % v/v ethanolic MPTMS solution). The ratio (A9-DNA:MPTMS) in the resulting reaction mixture is approx. (1:1), reaction time needed is 60 min, and then (A9-DNA-Au-NR-MPTMS) are obtained.

Fourth step is the attachment of hybridized NR to the microresonator. Values of FW and Q for the WGM mode before starting the whole experiment (milliQ water) should be in the range of 1-3 10^{-5} and 0.7 respectively, otherwise NR attachment leads to a loss of sensitivity that does not allows to proceed further. Replace 12 µL of milliQ water with citrate buffer 500 mM pH 3 in the 300 µL total cell volume. The mode will shift and within 20 min should reach a new equilibrium, pH 3 buffer causes the silica surface to become neutral or slightly positively charged thus increasing affinity towards NR. Add 10 µL of NR solution (concentration in the cell 6.4 pM), and within 5-10 min steps and spikes should appear, with a frequency of 10 events per min approximately. Wait until 10-20 steps which result in FW about 5 10^{-5} and Q no greater than 0.9 and wash with milliQ to check whether those values are maintained (and thus NR are still attached to the resonator, check diffraction spots in the camera, see the video). This procedure should render covalently bonded NR to the microresonator surface, but to achieve fully extended DNA strands, proper pH and ionic strength must be provided for a reasonable time. The microresonator could be left overnight in Hepes 30 mM pH 7.4 buffer and used the next day (minimum exposure to HEPES buffer before attempting C12 complementary DNA binding events should be 60 min).

Last stage of DNA binding experiments were carried using C12 solutions (full complementary of A912mer). Dilution is needed, 1:1 (100 µM) and 1:10 (10 µM) dilutions must be prepared with TE buffer. Adding 5 µL of C12 10 µM solution to the 300 µL HEPES buffer stabilized cell (approx. 150 nM C12 concentration) containing the hybridized microresonator should yield an increasing number of binding events (both spikes and steps) for increasingly higher ionic strengths (as additions of NaCl 500 mM solutions are made, in our case we replaced 20 µL of cell volume and wait for stable signal for about 5-10 min).

3) WGM experimental setup and resonators fabrication

The photonic-plasmonic whispering gallery mode (WGM) microsensor setup used for the experiments of Au-NR covalent binding was assembled with single-mode optical fiber. The glass fiber is first stripped of a protective coating and cleaned thoroughly with acetone and isopropanol to obtain a clean glass fiber. Then, a 30 W CO_2 laser, $\lambda \approx 10.6$ µm (Synrad 48-2, Novanta Inc., WA, USA), is used at 10 − 15% peak power to melt the glass fiber into a sphere using a home-built setup. The average final diameter of the spherical glass resonator is 80 – 90 µm.

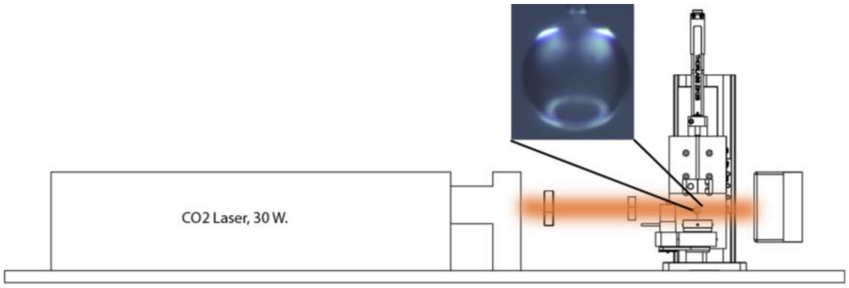

Figure S3.a. Schematic of the home-built setup setup to fabricate microspheres.

Silica fabricated microspheres were immediately used after prepared to avoid contamination. The as-produced microsphere was then mounted on the prism-based setup, in which WGM modes were excited using a fiber-coupled tunable external laser (Toptica, 642 nm) with beam diameter approx. 1 mm. The sampling cell in which the sensor is

immerser consist of a v-shaped chamber cut from polydimethylsiloxane (PDMS) with a cavity of 300 ml volume in which all in-situ described procedures were performed.

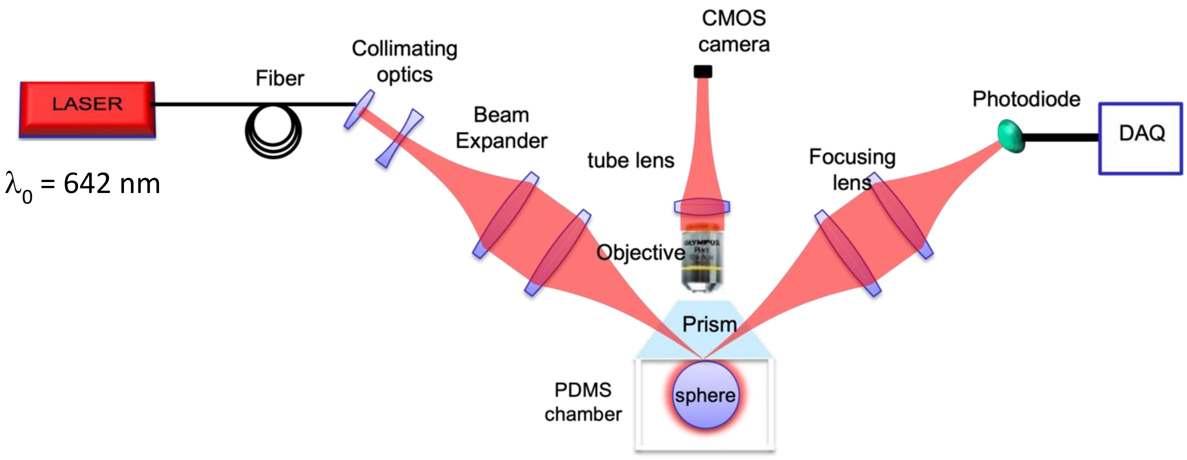

Figure S3.b. Schematic of the prism based microcavity sensor setup

4) Details of peak detection from WGM time series

Events of PNP interaction with the microresonator are evidenced as steps or spikes in both time series corresponding to Dk and Dl (always located in the upper and lower panel respectively). Below an example of such interacting PNPs and background (uneventful). Images of interference patterns observed in the microsphere upon irradiation with laser are also recorded, as interaction with PNPs can be detected as flashing features (see additional video and image below).

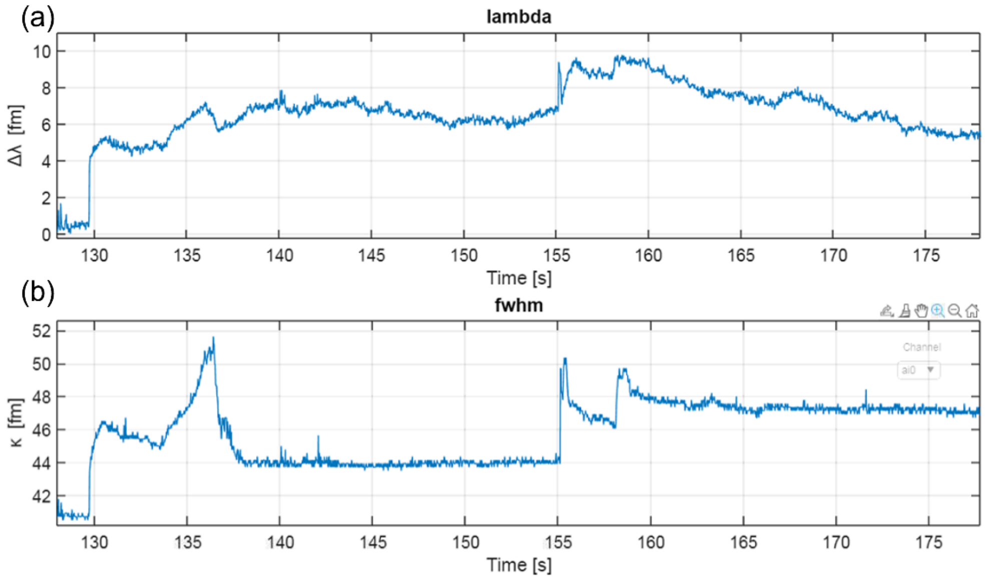

Figure S4. (a) (b) Time series for the variation of wavelength position (Dl) and linewidth (Dk) obtained in typical experiments carried using WGM microresonators exposed to colloidal suspensions of 28 pM Au-NR@HS-Si(OH)$_n$ (pH = 7 aqueous solutions).

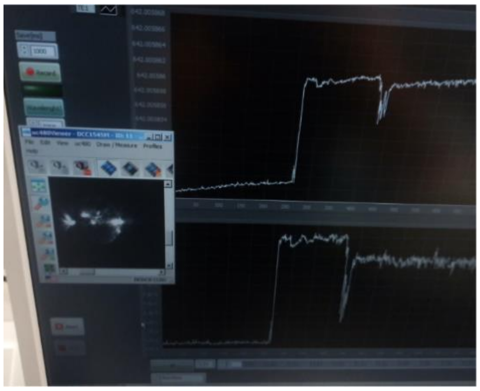

See suppl. Video provided corresponding to real-time interaction binding events between PNPs and resonator.

5) Poissonian statistics of single molecule interaction events

The Poisson distribution is a discrete probability distribution describing the probability of observing N “rare” events of interest within a fixed time interval. Underlying the Poisson

distribution is the assumptions that within a given infinitesimal time interval the probability of two events occurring is negligible, with only one or zero events being possible, in addition to the assumption of statistical independence of event occurrences between non-overlapping time intervals. Closely related to the Poisson distribution for the number of observed events (in our case binding or transient events) is the exponential distribution, which describes the statistics of the waiting time between events obeying Poisson statistics. To verify the single molecule nature of our observed events, we have thus performed a statistical analysis on the time intervals between observed interaction events, in which we find very close agreement with the expected exponential statistics for single molecule events. As an example, figure S5 are typical of fits performed on our datasets, in particular corresponding to events extracted from time series of Dk and Dl to 28 pM Au-NR@HS-Si(OH)n concentration.

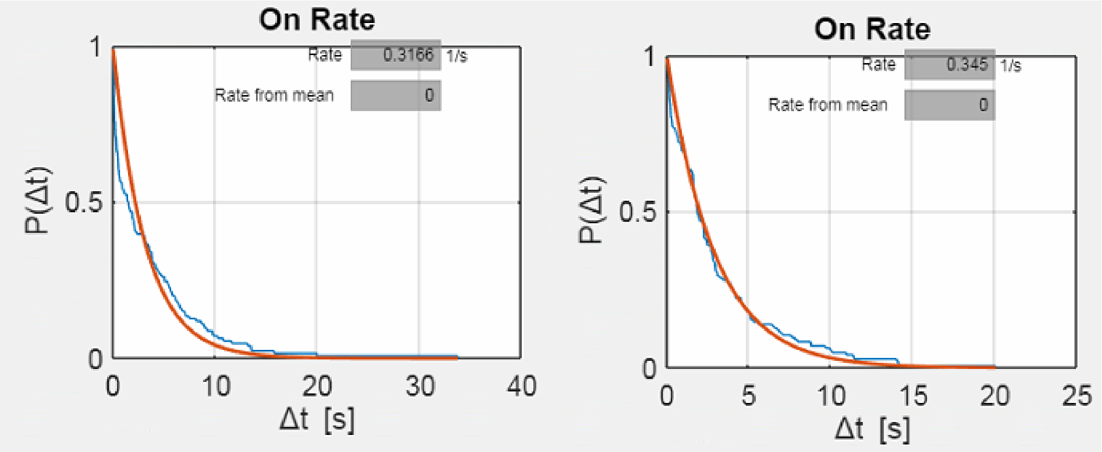

Figure S5. Statistics on time intervals between observed interaction events for 28 pM Au-NR@HS-Si(OH)n concentration: exponential decay constant for (a) Dk = 0.317 s ± 0.03 s and (b) Dl = 0.345 s ± 0.02 s

6) Brownian Motion

Increasingly higher event rates observed for in WGM time series as the concentration of PNPs increases. Assuming a Brownian behaviour for diffusing particles, the event rates can be calculated in principle. The number of PNP hits on an area after time t is described by

$$N = c \cdot A \cdot (D \cdot t)^{1/2}$$

where c is the PNP concentration, A the available surface on the resonator for interaction, and D the diffusion constant of PNPs. The latter is described by the Einstein-Stokes law

$$D = k_B \cdot T/(6 \cdot \pi \cdot \eta \cdot r) = \mathbf{1.8\ 10^{-11}\ m^2/s}$$

(Boltzman constant k_B, temperature T, viscosity η, PNP radius r).

7) Gold nanorods control experiments

Supplementary Figure S6-(a) shows a WGM time trace after addition of ~ 10 pM of nanorod without modification of MPTMS. No signals are observed during more than 20 min indicating the absence of detectable unspecific interactions between the resonator and the non-functionalized gold nanorod. Supplementary Figure S6-(b) shows that no significant WGM shift signals are observed after adding 500 mM of salt and buffer HEPES pH 7. For this experiment, the nanorod was modified with A9 oligonucleotide receptor.

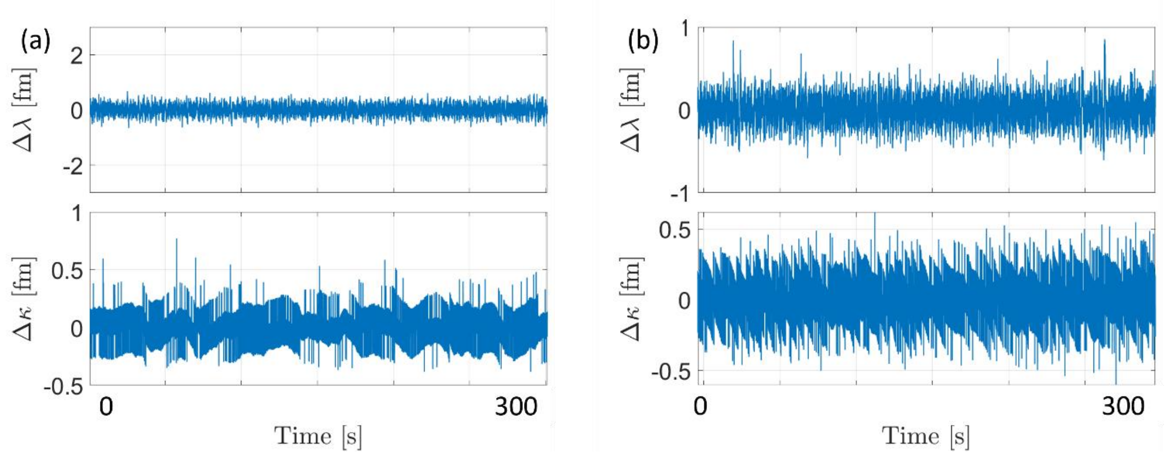

Figure S6. Time series for the variation of wavelength position ($\Delta\lambda$) and linewidth ($\Delta\kappa$) obtained using WGM microresonators exposed to **(a)** colloidal suspensions of gold nanorods without MPTMS treatment (pH = 7 aqueous solutions). **(b)** WGM microresonators after binding of Au-NR@HS-$Si(OH)_n$ then exposed to ionic strength of 500mM Na^+.

Supplementary Figure S7 shows that no significant WGM shift signals are observed under extreme basic condition, pH 11.5. For this experiment, the nanorod was modified with MPTMS, after PNP binding experiments the solution was removed from de chamber and washed five times with water. Then the pH was changed to 11.5 by adding a concentrated NaOH solution. No significant unbinding events of the nanorods were observed, indicating a very good stability under these experimental conditions.

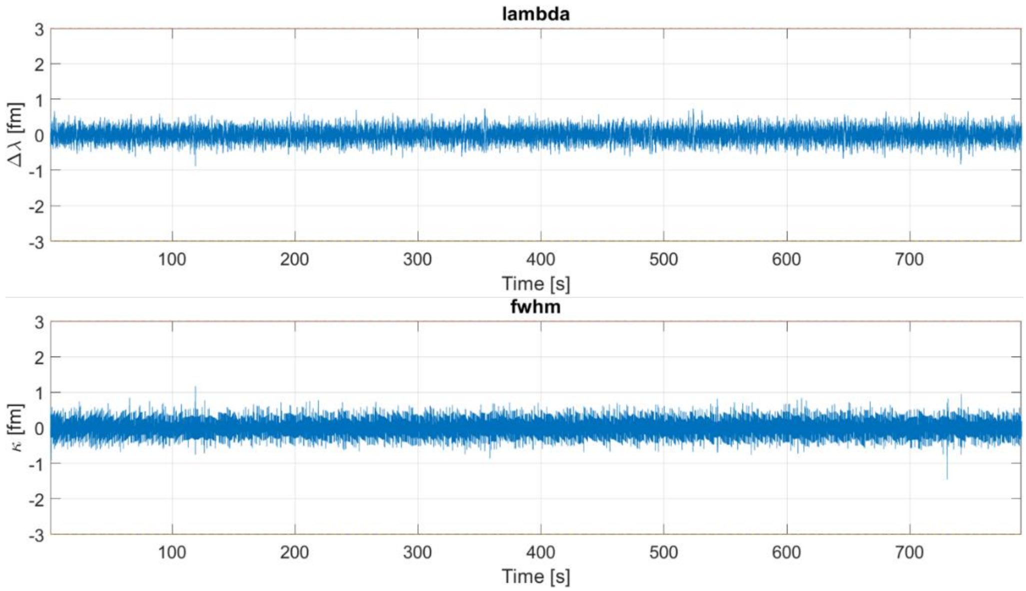

Figure S7. Time series for the variation of wavelength position ($\Delta\lambda$) and linewidth ($\Delta\kappa$) obtained using WGM microresonators after binding of Au-NR@HS-Si$(OH)_n$, then exposed to basic conditions (pH = 11.5 aqueous solutions).

8) Silanization activation energy calculations via Arrhenius equation

Histograms employed to obtain spike and step rate constants used for activation energy calculation within Arrhenius approximation.

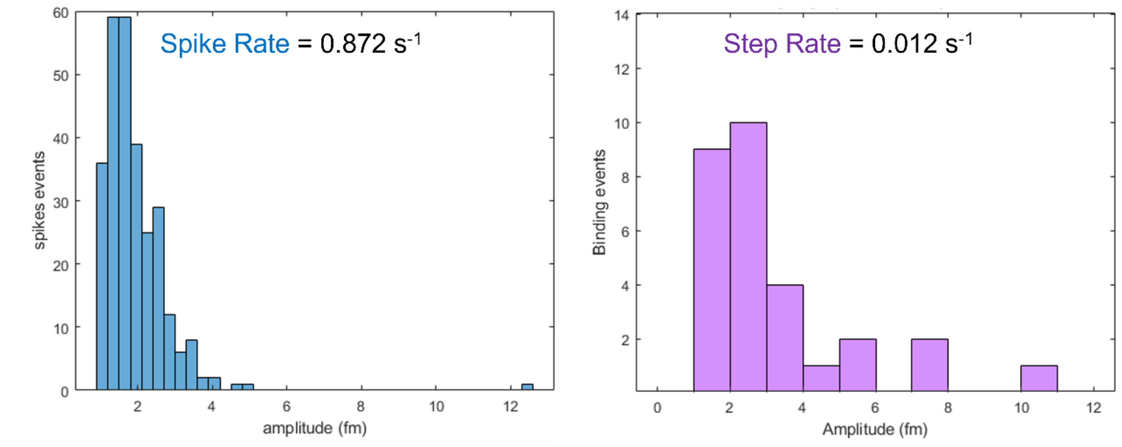

Figure S8. Histograms corresponding to events extracted from time series of Δκ corresponding to spike and step events observed for 2 pM of Au-NR@HS-Si(OH)$_n$ and 15 mM pH 3 citrate buffer.

9) **Nucleic acids interactions concentration scaling of event rate experiments**

Experiments were also performed to investigate the concentration dependence of observed transient events. We have determined the average rate directly from the time trace of wavelength shift measurements by determining the average rate of spike events due to single molecular interactions of a full complementary single DNA strand (C12) at low salt concentration (100 mM NaCl, 10mM buffer HEPES pH 7.4). Specificity of the signal follows since the unrelated DNA sequence does not produce any significant WGM shift signals (spike events) in control experiments, **Figure S9**.

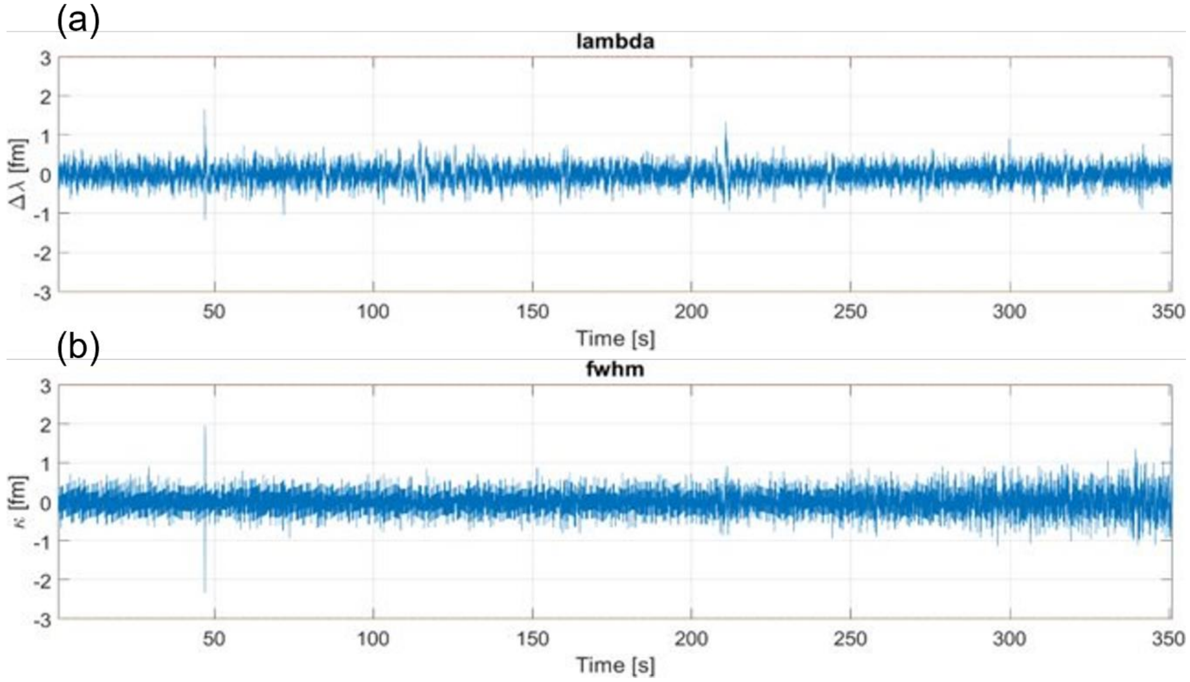

Figure S9. Time series for the variation of wavelength position ($\Delta\lambda$) and linewidth ($\Delta\kappa$) obtained using WGM microresonators after binding of Au-NR@HS-Si$(OH)_n$, then exposed to unrelated 12mer single stranded DNA (100 mM NaCl, 10 mM pH=7.4 HEPES buffer).

Linear fitting of the data yields a slope of 2.22×10^6 $M^{-1}s^{-1}$ (R^2 = 0.995), such that, considering an average of 21 receptors (as determined from counting subsequent binding events observed upon NaCl 250 mM salt concentration, Figure S10).

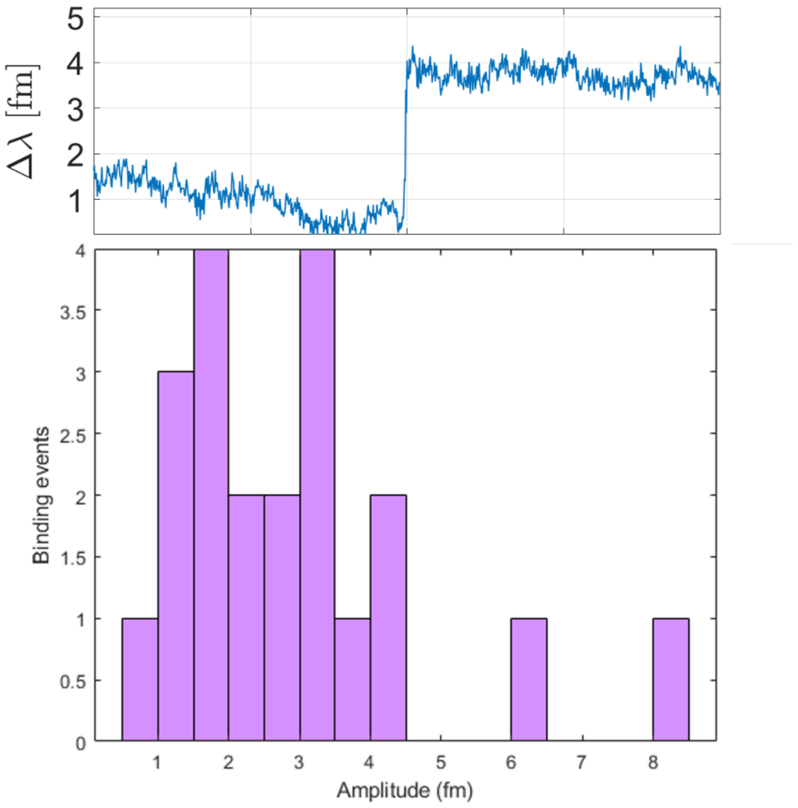

Figure S10. Time series for the variation of wavelength position ($\Delta\lambda$) obtained using WGM microresonators after binding of Au-NR@HS-Si(OH)$_n$, steps signals after adding 166 nM of C12 full complementary and 250 mM of NaCl. Histograms corresponding to binding events extracted from time series of $\Delta\lambda$.
